# Mechanical regulation of cardiac development

**DOI:** 10.3389/fphys.2014.00318

**Published:** 2014-08-21

**Authors:** Stephanie E. Lindsey, Jonathan T. Butcher, Huseyin C. Yalcin

**Affiliations:** ^1^Department of Biomedical Engineering, Cornell UniversityIthaca, NY, USA; ^2^Department of Mechanical Engineering, Dogus UniversityIstanbul, Turkey

**Keywords:** hemodynamics, congenital heart defects, heart development, mechanotransduction, shear stress

## Abstract

Mechanical forces are essential contributors to and unavoidable components of cardiac formation, both inducing and orchestrating local and global molecular and cellular changes. Experimental animal studies have contributed substantially to understanding the mechanobiology of heart development. More recent integration of high-resolution imaging modalities with computational modeling has greatly improved our quantitative understanding of hemodynamic flow in heart development. Merging these latest experimental technologies with molecular and genetic signaling analysis will accelerate our understanding of the relationships integrating mechanical and biological signaling for proper cardiac formation. These advances will likely be essential for clinically translatable guidance for targeted interventions to rescue malforming hearts and/or reconfigure malformed circulations for optimal performance. This review summarizes our current understanding on the levels of mechanical signaling in the heart and their roles in orchestrating cardiac development.

## Introduction

The heart is the first functional organ to develop in the embryo, convecting nutrients to surrounding tissues to facilitate growth. As the embryo grows, the heart transforms from a linear valve-less tube to a multi-chambered structure complete with 4 fibrous valves (Srivastava and Olson, 2000; Bartman and Hove, 2005). Changes in pressure, strain and wall shear stress (WSS) accompany cardiac morphogenesis and orchestrate molecular and cellular responses that help coordinate downstream tissue changes. Congenital heart defects (CHDs) form when cardiac morphogenetic processes are disrupted. CHDs affect 1–2% of newborn children and are the leading cause of death in infants under 1 year of age. CHDs represent the single largest class of birth defects and account for approximately 25% of all human congenital abnormalities (Roger et al., 2011). Despite their prevalence, the etiology of many CHDs remains unknown. While clinical and experimental research has identified multiple genetic mutations that participate in the formation of CHDs, they fail to fully account for the disease phenotype. Extreme locus heterogeneity and lack of a distinct genotype–phenotype correlation have limited causative gene discovery (Yuan et al., 2013). Recent insights into the molecular mechanisms of heart development have shown that a given structural CHD is often linked to multiple loci. A variety of phenotypes is often observed in families with a specific gene mutation (Nemer, 2008). Family members with the same mutation may present with an atrial septal defect, tetralogy of fallot, or ventricular septal defect (Bruneau, 2008). Conversely, mutations in different genes may cause an identical malformation (Fahed et al., 2013). Zaidi and colleagues recently identified new point mutations in hundreds of genes that together may contribute to only approximately 10% of CHDs (Zaidi et al., 2013). Oyen et al. determined the risk of an infant presenting with a CHD in families with prior history of CHDs was only 2–4%, suggesting CHDs largely occur in families without history of disease (Oyen et al., 2009). In addition to genetic factors, environmental factors such as drug exposure and hemodynamic patterning contribute to the development of CHDs (Huang et al., 2010). Mechanical perturbation of blood flow can induce diseased phenotypes (Hogers et al., 1999; Sedmera et al., 1999, 2002; Miller et al., 2003; Reckova et al., 2003; Groenendijk et al., 2005; deAlmeida et al., 2007).

**Figure 1:**
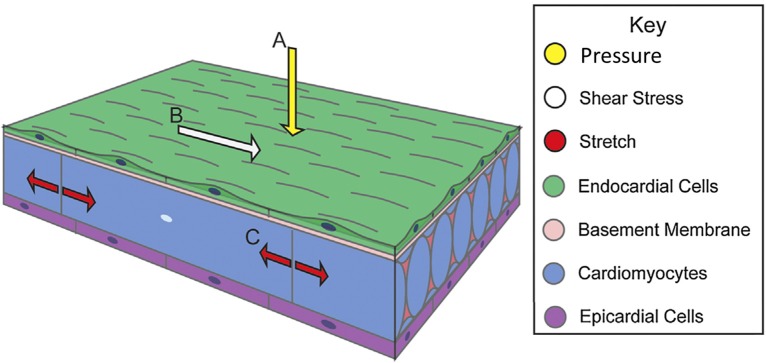
**Biomechanical forces in cardiac wall maturation**. Biomechanical forces are important for normal developmental patterning. Forces exerted on the wall from blood flow include (A) pressure, a force perpendicular to the vessel wall, (B) shear stress, the frictional force parallel to the vessel wall, and (C) circumferential stress that occurs as the walls stretch. Arrows indicate force vectors. Adapted from Samsa et al. (2013).

Blood flow exerts forces on surrounding tissue. These forces include, the force normal to the walls from blood pressure, the associated circumferential stress that occurs as the walls stretch in response to pressure and the frictional force exerted by flow along the walls, WSS (Gjorevski and Nelson, 2010; Samsa et al., 2013) (Figure 1). Local mechanical properties of cardiac tissue as well as its loading-induced residual stress govern structural deformation in response to mechanical stimuli. Mechanical signals induce gene expression and differentiation on a cellular level, translating molecular level events into tissue-level deformations that guide embryo development (Wang et al., 2009; Mammoto and Ingber, 2010; Yalcin et al., 2011; Bharadwaj et al., 2012; Wyczalkowski et al., 2012). This review summarizes cardiac morphogenesis and the role of mechanical signaling in its effectuation.

## Basic steps of heart development

**Figure 2:**
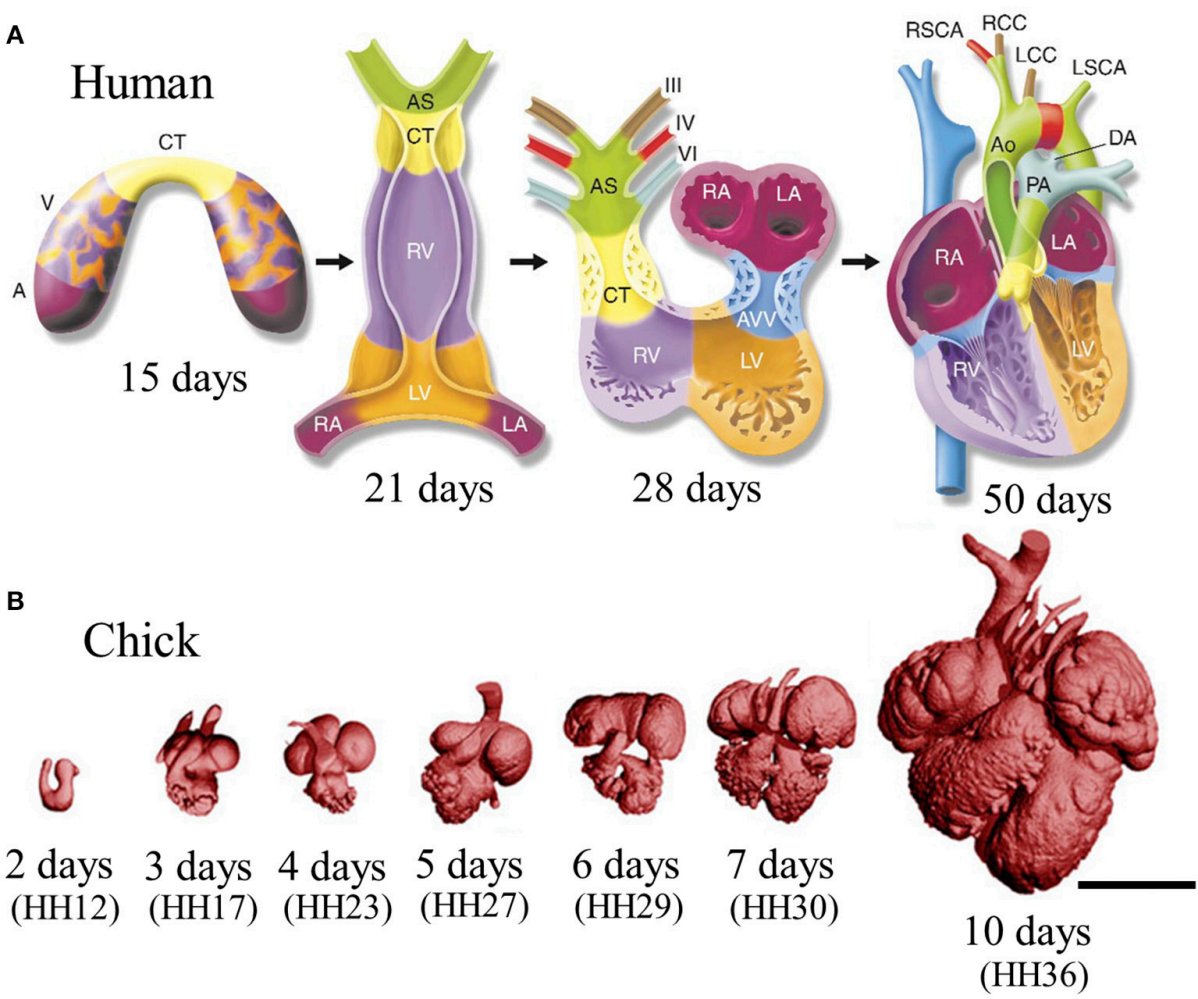
**Stages of heart development. (A)** Schematic of cardiac morphogenesis in human. Illustrations depict cardiac development with color coding of morphologically related regions, seen from a ventral view. Cardiogenic precursors form a crescent (far-left panel) that is specified to form specific segments of the linear heart tube, which is patterned along the anteroposterior axis to form the various regions and chambers of the looped and mature heart. Each cardiac chamber balloons out from the outer curvature of the looped heart tube in a segmental manner. Neural crest cells populate the bilaterally symmetric aortic arch arteries (III, IV, and VI) and aortic sac (AS) that together contribute to specific segments of the mature aortic arch. Mesenchymal cells form the cardiac valves from the contruncal (CT) and atrioventricular valve (AVV) segments. Corresponding days of human embryonic development are indicated. Abbreviations: A, atrium; V, ventricle; RV, right ventricle; LV, left ventricle; RA, right atrium; LA, left atrium; PA, pulmonary artery; Ao, aorta; DA, ductus arteriosus; RSCA, right subclavian artery; LSCA, left subclavian artery; RCC, right common carotid; LCC, left common carotid. Adapted from Srivastava and Olson (2000). **(B)** Scaled micro-CT images of chicken embryonic hearts at representative days. Corresponding Hamburger–Hamilton (HH) stages were shown in paranthesis. Images were generated by our group in our previous work. Scale bar is 1 mm.

While cardiac morphogenesis varies across vertebrate species, major landmarks can be broken down into four basic steps, namely, heart tube formation, looping, trabeculation and valve formation/septation. Major developmental events are summarized below as well as in Table 1 and in Figure 2. Tables 2, 3 evaluate changes in heart rate and blood pressure in humans and in common, animal models of cardiovascular development.

**Table 1:** **Comparative cardiovascular development across animal models**.

**Human [weeks]**	**Mouse (E)**	**Chick (HH)**	**Zebrafish (hpf)**	**Major events in heart development**
22 [3 weeks]	7–8	7–10		Fusion of paired heart tubes
22 [3 weeks]	7.5–8.5	10	24–36	First appearance of myofibrils in myocytes
				First myocardial contractions
				Cardiac looping (mouse E 8.5)
24 [3.5 weeks]	8–8.5	9–12+	22	First blood flow through heart
26 [3.5+ weeks]	9–11	11–12		First ventricular trabeculations
28 [4 weeks]	10–12	13–22	60	First definable endocardial cushions (chick 28)
29 [4 weeks]	11–13.5	15–23		First appearance atrial septum prium
31 [4.5 weeks]	12	24–28		First appearance primordial semilunar valves, start AV septation
33 [4.5+ weeks]	12–13	25–28	96	Completion AV septum
35 [5 weeks]	13–15	26–31		Completion intraventricvular septation
37–43 [5+ weeks to 6 weeks]		27–34	105	Maturation semilunar valves

*Mouse staging is based on embryonic day (E), chick is Hamburger–Hamilton staging (HH), zebrafish is hours post-fertilization (hpf). Adapted from Lindsey and Butcher (2011)*.

**Table 2:** **Heart rate (HR) in beats per minute (bpm) across developmental animal models**.

**Human**	**Days or weeks**	**HR, bpm**	**Zebrafish**	**dpf**	**HR, bpm**
	37 days	101–109		2	141
	41 days	120–134		3	147.2
	45 days	130–158		4	165.9
	50–52 days	120–175		5	171.5
	8+ weeks	150–176			
	9+ weeks	150–172			
	10+ weeks	140			
	11+ weeks	140			
	12+ weeks	155–158			
**Chick**	**HH**	**HR, bpm**	**Mouse**	**emb day**	**HR, bpm**
	16	110		10.5	124.7
	18	147.5		11.5	135.6
	21	145		12.5	147.3
	24	155		13.5	173.6
	27	155		14.5	194.3
	29	194		15.5	209
	31	221			
	35	230			

*Mouse staging is based on embryonic day (emb day), chick is Hamburger–Hamilton staging (HH), zebrafish is days post-fertilization (dpf). Adapted from Lindsey and Butcher (2011)*.

**Table 3:** **Blood pressure across developmental animal models**.

**Human**	**Weeks**	**Right ventricular systolic pressure (mmHg)**	**Right ventricular diastolic pressure (mmHg)**	**Zebrafish**	**Body mass (mg)**	**Systolic pressure (mmHg)**	**Diastolic pressure (mmHg)**
	14	30.99	8.45		0.5	0.06	0.35
	16	35.42	9.65		1.25	0.10	0.84
	18	39.85	10.86		2.25	0.16	1.49
	20	44.28	12.07		3.25	0.22	2.14
	22	48.70	13.28		3.75	0.29	2.47
	24	53.13	14.48				
	26	57.56	15.69				
**Chick**	**HH**	**Ventricular systolic pressure (mmHg)**	**Ventricular diastolic pressure (mmHg)**	**Mouse**	**emb day**	**Systolic pressure (mmHg)**	**Diastolic pressure (mmHg)**
	16	1.15	0.25		10.5	3.44	0.52
	18	1.31	0.33		11.5	5.01	0.50
	21	1.61	0.34		12.5	6.43	0.90
	24	1.96	0.4		13.5	9.0	0.86
	27	2.35	0.56		14.5	11.15	0.88
	29	3.45	0.82				

*Mouse staging is based on embryonic day (emb day), chick is Hamburger–Hamilton staging (HH). Adapted from Lindsey and Butcher (2011)*.

During early stages of cardiogenesis, two bilateral mesodermal heart forming fields, also known as the primary heart fields (PHF), fuse at the ventral midline, to form the tubular heart (Rana et al., 2013). At this stage, the cardiac wall is composed of an inner endocardial layer and outer myocardial sheath. Separating these two layers is a gelatinous acellular hyaline matrix called the cardiac jelly. The cells in this newly formed linear heart tube will primarily contribute to the developing atria, atrioventricular (AV) canal, and the left ventricle (Meilhac et al., 2004; Zaffran et al., 2004).

Expansion and elongation of the heart tube initiates the next stage of cardiac formation: looping. Elongation begins with the addition of cardiac progenitor cells to either end of the tubular heart. This population of highly proliferative mesodermal tissue is commonly called the second heart field (SHF) (Dyer and Kirby, 2009). At the arterial pole, these cells will contribute to the outflow tract (OFT), right ventricle, and the interventricular septum and at the venous pole, they contribute to the developing atria and the atrial septum (Verzi et al., 2005; Lockhart et al., 2011). Looping occurs in three phases: dextral looping or c-looping, formation of the primitive s-loop, and formation of the mature s-loop (Manner, 2004). Asynchronous contractions begin with the formation of the linear tubular heart, but become coordinated once looping starts to propel blood (Goenezen et al., 2012). At the C-loop stage, the once straight heart tube becomes a looped structure with a morphologically distinct primitive atrium, primitive ventricle, and primitive OFT. During S-looping, regional wall thickenings of cardiac jelly form endocardial cushions (ECs) in the AV canal and OFT regions. At this stage, ECs function as primitive valves by closing off the lumen during contraction, facilitating unidirectional pulsatile flow (Butcher et al., 2007).

Ventricular segment trabeculation is initiated toward the last stages of looping. During cardiac trabeculation, cardiac jelly is displaced from the ventricle's outer curvature and endocardial extensions grow toward the myocardial layer (Moorman and Christoffels, 2003). These extensions then form a network of luminal projections called trabeculae. Trabeculae consist of myocardial cells covered by an endocardial layer (Samsa et al., 2013); greatly increasing surface area, as well as myocardial mass, and wall stiffness (Yang et al., 1994). The increase in surface area boosts nutrition and oxygen uptake in the embryonic myocardium prior to coronary vascularization without increasing heart size. This enhancement leads to an increase in cardiac output as well (Liu et al., 2010). Trabecular compaction then follows, and contributes to the formation of the ventricular septum and to the thickening of the ventricular compact layer (Goenezen et al., 2012). As the compact layer grows in size and complexity, it replaces trabeculae as the major contractile force. Compact myocardium ultimately provides most of the myocardial mass and therefore contractile force in the mature heart (Wessels and Sedmera, 2003). During chamber growth, the ventricular conduction system differentiates as well. This differentiation is marked by an apparent reversal in the sequence of ventricular activation (Chuck et al., 1997). The immature base-to-apex pattern of epicardial activation switches to a mature apex-to-base pattern (Gourdie et al., 2003).

Septation of the atrium and the ventricle transforms the heart into a four-chambered organ. Septation occurs via trabecular compaction and the fusion of ECs (van den Berg and Moorman, 2009). ECs are protrusions inside the AV canal and OFT and work as primitive valves, performing a sphincter-like function until mature valves develop (Butcher et al., 2007). ECs consist of mesenchymal progenitor cells with overlying endocardial endothelial cells. The EC mesenchyme is derived from the AV canal and OFT endocardial endothelial cells after an endothelial-to-mesenchymal transition (Person et al., 2005), with minor and transient contributions from epicardial or neural crest derived cells. The AV ECs fuse and condense into mitral and tricuspid valve leaflets whereas the distal OFT ECs are remodeled into the semilunar valve leaflets (Combs and Yutzey, 2009).

## Biophysical mechanisms of heart tube formation

The underlying mechanics of heart tube formation is not fully understood. Extracellular matrix (ECM) fiber interactions and cell adhesions are important early mechanical stimuli. Through fibronectin and mesodermal cell tracking, Zamir et al. showed that convective tissue motions contribute significantly to total cell displacement (Zamir et al., 2006). Cell autonomous displacements (i.e., cell movement relative to the ECM) decreased gradually after egression from the primitive streak, with caudual cells actively moving faster than cranial cells. The tracking of ECM tags relative to endocardial progenitors, however, revealed little active motility of endocardial cells relative to its surrounding ECM (Aleksandrova et al., 2012). In a study that investigated migration of the bilateral heart fields to the embryonic midline, Vamer et al. showed that *endodermal shortening* around the anterior intestinal portal is responsible for movement of cardiac mesoderm and endoderm toward the midline (Varner and Taber, 2012). Endodermal shortening is driven by cytoskeletal contraction, and can be arrested by blebbistatin, a myosin-II inhibitor. Shortening was shown to decrease both tissue stiffness and mechanical tension. These findings highlight the importance of large-scale tissue deformations in transporting cells to their appropriate positions during heart tube formation.

As a cell engages ECM and actively applies force on its surrounding matrix, it senses local elastic resistance of the ECM and nearby cells (Buxboim et al., 2010). ECM or tissue elasticity has an influential role in regulating cell functions such as migration and proliferation (Hadjipanayi et al., 2009; Winer et al., 2009). Cell migration and proliferation are modulated by cell-generated, actin-myosin forces that both depend on and influence matrix elasticity. Embryonic tissue elasticity may therefore be more strongly influenced by cellular cytoskeleton than in adult. Forces exerted on the cell by the ECM may affect the expression of matrix-sensitive genes (Buxboim et al., 2010). Cells can also respond to the stiffness of their surrounding matrix. For example, cardiomyocyte contraction and myocyte architecture development is optimized on substrate elasticity mimicking that of the developing myocardial environment (Engler et al., 2008; Majkut et al., 2013). This data suggests that, cell-ECM interactions are important to the placement of cardiac progenitor cells and their subsequent differentiation during cardiogenesis.

Forces initiating the furling of cells into the primitive heart tube are not well-understood. One hypothesis is that the cylindrical bending of a sheet of epithelial cells could result in the formation of a hollow tube (Taber, 1998). The key to this theoretical model is the asymmetric contraction of actin microfilaments surrounding the apices of the epithelial cells, causing the normally cylindrical cells to become more wedge shaped. This shape change eventually forces the apical surface of the sheet into the inner curvature of the developing tube. The ability of individual cells to resist apical–basal elongation during this contraction would result in a more substantial bending of the sheet (Bartman and Hove, 2005). Confocal laser scanning microscopic studies have revealed that the early cardiac epithelial cells do in fact have such a microfilament arrangement (Shiraishi et al., 1992), and pharmacological inhibition of the microfilament results in failed heart tube formation (Ettensohn, 1985).

## Initiation of the heart beat

Spontaneous action potentials can be detected in the newly formed chick heart tube at Hamburger–Hamilton (HH) stage 9 before myocyte contraction begins (Kamino et al., 1981). Starting around early HH10, the tubular heart beats irregularly and slowly without effective blood flow. By the end of stage HH10, a clear heart rhythm is present (Hogers et al., 1995). The frequency of this rhythm increases and blood flow can be detected as early as HH11 (Granados-Riveron and Brook, 2012). Effective blood flow starts during looping at HH12 and at which time a peristaltic wavelike contraction pattern is observed along the length of the heart tube, from the atrium to the OFT (Taber, 2006). By HH20, a vigorous circulation is established (Granados-Riveron and Brook, 2012).

One would think that the embryonic heart starts beating in order to pump blood for convective transport. However, chicken embryos do not show hemoglobin-mediated transport of oxygen until HH20 (Cirotto and Arangi, 1989). It can therefore be concluded that diffusion is a sufficient means of transport for oxygen, nutrients, metabolic wastes, and hormones in the early chick embryo (Burggren, 2004). Total elimination of cardiac ejection by complete ligation of the cardiac OFT in HH20–HH23 chick embryos has no significant effect on O_2_ consumption in the 4 h after surgical manipulation (Burggren et al., 2004). If it is not the nutrition transport, than what is the purpose of the initial heart beat?

Although the role of early cardiac contraction is unclear, recent evidence suggests that hemodynamics generated by contraction of cardiomyocytes acts to drive cardiogenesis on a molecular and cellular level (Granados-Riveron and Brook, 2012). Lucitti et al. showed that lower hematocrit results in lower shear stress and defective vessel remodeling in the embryonic mouse (Lucitti et al., 2007). These defects were subsequently rescued through the resotration of control viscosity. These results support that hemodynamic force is necessary and sufficient to induce vessel remodeling. To study the effects of hemodynamic forces on heart development, flow was occluded at either the cardiac inflow or OFT of zebrafish embryos [37 hours-post-fertilization (hpf), straight heart tube stage] in order to reduce shear stress in the AV canal (Hove et al., 2003). Inflow and outflow obstruction resulted in hearts with an abnormal third chamber, diminished looping and impaired valve formation at later stages. Hove et al. stipulate that the observed cardiac abnormalities were the result of altered intracardiac flow patterns that reduced local WSS acting on the endocardial cells. Bartman et al. investigated the respective roles of *myocardial function* (contraction) and blood flow induced *shear stress* in EC development for zebrafish embryos (Bartman et al., 2004). In their experiments, they varied the degree of myofibril inhibition using different concentrations of 2,3-butanedione monoxime and studied the resulting effects on blood flow. Myocardial function decreased in dose dependent manner with myofiber inhibition, and the percentage of embryos that formed endocardial rings at 48 hpf also decreased. Interestingly, at very high concentrations, blood flow was completely abolished and no shear stress existed though myocardial function persisted, with 58% of these embryos still forming an endocardial ring. These studies did not assess the potential for changes in secreted molecule profile from the myocardium, but the results support that both shear stress and myocardial function play a role in cardiogenesis (Mironov et al., 2005).

## Biophysical mechanisms of looping

Manning and MacLachlan showed that isolated tubular hearts could bend but not rotate during the c-loop phase of cardiac development (Manning and McLachlan, 1990). Their results suggest the bending component of C-looping is intrinsic to the heart tube, while torsion is mainly driven by extrinsic forces (Wyczalkowski et al., 2012). Actin polymerization-driven myocardial cell shape changes have been found to contribute to the bending of the heart tube (Manasek et al., 1972; Latacha et al., 2005). The torsional component of C-looping is largely due to forces from its encapsulating membrane, the splanchnopleure (SPL) (Taber et al., 2010). When the SPL is removed from HH11 chick hearts, they no longer maintain their looped configuration (Voronov and Taber, 2002; Voronov et al., 2004). External forces may not be alone in guiding cardiac rotation. When the SPL is removed from embryonic chick hearts around the onset of looping (HH10), torsion is initially suppressed, but restored several hours later (Nerurkar et al., 2006). Delayed torsion was found to coincide with increased myocardial stiffness, suggesting that delayed torsion is caused by an abnormal cytoskeletal contraction. Overall, these results show that while intrinsic cytoskeletal forces contribute to the dextral bending component of C-looping, both intrinsic and extrinsic forces are required for the rotational component of C-looping.

## Foce generation of the tubular heart

In the tubular heart, cyclic generation of traveling mechanical waves sweep from the venous to arterial end generating a unidirectional blood flow. These traveling mechanical waves were traditionally considered myocardial peristaltic waves (Xavier-Neto et al., 2007) and tubular embryonic heart was accepted to work like a technical roller peristaltic pump (Manner et al., 2010). Experiments on zebrafish embryos offer an alternative theory (Forouhar et al., 2006): (I) While peak flow velocity generated by a roller peristaltic pump corresponds to the speed of a compression wave, peak ventricular inflow velocity of an embryonic zebrafish heart recorded exceeds the speed of a traveling contraction wave. (II) In the zebrafish embryo, the relationship between cardiac contraction frequency and the flow rate is non-linear and exceeds the maximum flow rate possible for a roller peristaltic pump. (III) Roller peristaltic pumps are marked by non-stationary sites of active compression that move in a uniform direction along the length of a flexible tube whereas the early embryonic zebrafish heart possesses a single stationary center of active myocardial contractions at its venous pole. Several other studies support these findings. The tubular embryonic heart does not function as a technical roller peristaltic pump (Hu and Clark, 1989; Butcher et al., 2007), but may be considered a valveless “Liebau pump” (also known as suction or impedance pump) (Manner et al., 2010). Valveless pumping can be achieved experimentally by periodically compressing the asymmetric site of a fluid filled tube made up of stiff and soft elastic sections (Liebau, 1955, 1956; Ottesen, 2003; Hickerson et al., 2005). Here, periodic compression at an asymmetric site in the soft elastic region leads to unidirectional flow. In order to be considered Liebau driven flow, the tube must have a flexible wall and a finite length with active compression stemming from a small asymmetric non-central section. Flow generated by Liebau pumps is typically pulsatile (Manner et al., 2010).

Forouhar et al. identified a single site of active myocardial contractions as well as a non-linear relationship between contraction frequency and flow rate in the embryonic zebrafish heart (Forouhar et al., 2006). They concluded that tubular embryonic hearts work as Liebau pumps rather than peristaltic pumps. Previous work by our group investigated pumping mechanisms at various stages of embryonic chick development (Butcher et al., 2007). At HH17 blood velocity exceeds that of tissue velocity, and wave propagation is initiated by a single myocardial source, supporting the Liebau pump hypothesis. At HH25 the embryo utilizes a piston-pumping mechanism. Piston pumping is defined as a volume change–driven propulsion of fluid, with the orifice working to throttle the outlet flow. HH21 embryos don't fall into one pumping category, but rather act as a transition stage between two pumping styles. This same transition can be seen in the pumping mechanism of zebrafish embryonic hearts at 36 hpf (Johnson et al., 2013).

Although the early embryonic heart does not function as a technical roller peristaltic pump, it still incorporates peristaltic mechanisms. Taber et al. showed that peristaltic heart tubes can generate pulsatile blood flow as a result of the presence of ECs at the inflow and outflow of the ventricular loop (Taber et al., 2007). Traveling contractile waves generate pressure and flow values an order of magnitude greater in tubes with valvular protrusions. In their peristalsis model with ECs, flow velocities exceeded the contractile wave speed by 75%. These results highlight the importance of ECs in pulsatile pump force generation.

## Biophysical mechanisms of chamber maturation

### Effects of mechanical factors on chamber development

**Figure 3:**
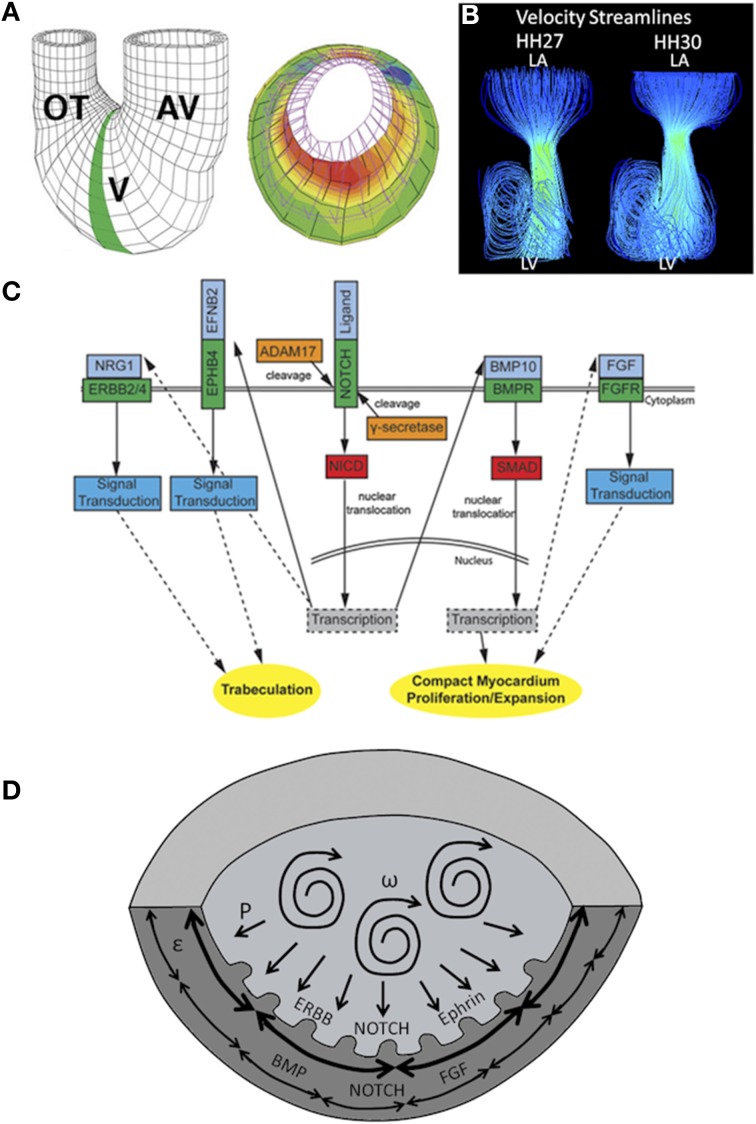
**Mechanical forces driving chamber development. (A)** A computational model showing that inner trabecular layers undergo greater strain that outer compact layer in ventricular chamber. AV, atrioventricular canal; V, ventricle; OT, outflow tract. Adapted from Damon et al. (2009). **(B)** Velocity streamlines in left AV canal for HH27 and for HH30 chicken embryos, generated with computational fluid dynamics. Strong vortices develop in the ventricular cavity, possibly necessary for the expansion of the chambers. Adapted from Yalcin et al. (2011). **(C)** Signaling pathways in ventricular chamber maturation. NOTCH, ERBB, and Ephrin play role in trabeculation whereas NOTCH, BMP, and FGF play role in compact myocardium development. Adapted from Samsa et al. (2013). **(D)** Proposed ventricular chamber development model. Circulatory flows cause expansion of the ventricles. Pressure induces a gradient in circumferential strain in the trabeculae and compact myocardium. This gradient cause a difference in myocyte proliferation as well as different gene expression profiles. P represents pressure, ω represents circulations, and ε represent circumferential strain.

Damon et al. measured a gradient of strain across the chamber wall, with strain greatest along inner layers and lowest in the compact myocardium (50% elongation vs. 20% elongation) (Figure 3A) (Damon et al., 2009). Cell proliferation was found to be greatest in the compact myocardium and lowest in the trabeculae (Sedmera and Thompson, 2011). A separate study using zebrafish embryos found that *erbb2*, a regulator of myocyte proliferation, contributes to ballooning and chamber growth during heart development (Liu et al., 2010). Together these results suggest strain magnitudes may alter proliferation rates, retarding proliferation with an increase in strain magnitude. Indeed WSS and strain play a major role in cardiogenesis. Many computational models modulate growth as a function of stresses and strains (Taber, 2009; Buskohl et al., 2012b; Rugonyi, 2013).

*Cell morphologies* have also been shown to contribute to the ballooning process of heart chambers. Ballooning involves the expansion and bulging of the linear walls of the looped heart tube into bean-shaped chambers, with a convex outer curvature and a concave inner curvature (Gjorevski and Nelson, 2010). High-resolution imaging of the developing zebrafish hearts revealed that cells of the outer curvature flatten and elongate relative to those of the inner curvature, which maintain a cuboidal shape. The characteristic curves of cardiac chambers are due to these differences in cellular morphologies (Auman et al., 2007). Given that the inner curvature of the early heart is exposed to higher WSS than the outer curvature (Hierck et al., 2008), one may suppose shear stress levels affect cellular morphologies at these locations. Employing zebrafish mutants with functional deficiencies, Auman et al. showed that in addition to blood flow forces, contractility also independently regulate cell shape changes in the emerging ventricle (Auman et al., 2007). Blood flow, an external force, promotes ventricular cell enlargement and elongation whereas contractility, an internal force, restricts ventricular cell size and elongation. Thus, the acquisition of normal cardiomyocyte morphology and therefore chamber morphology, requires a balance between extrinsic and intrinsic physical forces. Recently, Chi et al. showed that, independent of contractile and hemodynamic forces, as an intrinsic force, electrical forces may also affect cardiomyocyte morphology and subsequent chamber morphology (Chi et al., 2010).

Finally, previous work by our group showed that, in addition to shear stress levels and profiles, blood flow patterns evolve within developing hearts. Specifically, flow remains laminar in the HH17 heart, and after septation at HH27, jet flow in the AV canal allows strong vortices to develop in ventricular cavities. The emergence of these vortices coincides with ventricular expansion, suggesting vortex formation may affect chamber maturation (Figure 3B) (Yalcin et al., 2011). A summary of gene expression pathways that play a role in chamber maturation is given in Figure 3C. Figure 3D proposes a model of chamber development based on the aforementioned studies.

### Chamber maturation

In an effort to understand the contribution of hemodynamic forces on the development of CHDs, many animal models and experimental techniques have been developed. Two common models of flow manipulation in the chick embryo are conotruncal banding (CTB) and left atrial ligation (LAL). In CTB, a knot is tied around the conotruncus region of the OFT at HH18 in order to induce an outflow flow constriction (Clark et al., 1984). In LAL, the left atria is partially ligated at HH20–21, obstructing flow to the ventricle (Sedmera et al., 1999). CTB results in an immediate increase in pressure and is therefore considered an increased afterload model. LALs shift blood flow to the right side of the heart and are considered a model of increased right ventricular and decreased left ventricular preload (Sedmera et al., 1999).

Tobita et al. measured flow velocities in the AV canal and examined wall deformation patterns in the right and left ventricle (RV and LV, respectively) following LAL (Tobita and Keller, 2000). Decreases in max as well as average velocity were evident immediately following LAL. At HH31 (post-septation), average RV inflow velocity was found to be higher in LAL embryos, while maximum LV inflow velocity was lower. These results suggest flow is redirected to the right side of the heart after LAL. Increased right ventricular flow accelerated the onset of RV circumferential strain patterns, and decreased flow in LV abolished these patterns. Kowalski et al. investigated immediate alterations in blood flow patterns and WSS levels following LAL through the use of a computational fluid dynamics (CFD) model (Kowalski et al., 2014). Intracardiac flow patterns are immediately altered following LAL, resulting in a decrease in WSS levels at the left AV canal as well as at the left side of the common ventricle (Kowalski et al., 2014). WSS level decreased from 10 dynes/cm^2^ to 5 dynes/cm^2^ at these locations for LAL embryos. Using optical coherence tomography, Rugonyi et al. studied immediate changes in blood flow velocities and wall motions in the OFT region of HH18 chick embryos following CTB (Rugonyi et al., 2008). CTB resulted in an increase in the blood velocity in the banded part of the OFT due to flow constriction at that region. Strain patterns revealed that mechanically altered (banded) embryos exerted more energy to maintain growth.

Sedmera et al. investigated morphological abnormalities due to hemodynamic alterations resulting from LAL and CTB (Sedmera et al., 1999). Following CTB, a thickening and dilation of the compact myocardium and trabeculae in the LV was observed. LAL resulted in hypoplasia of the left heart structures, most likely due to decreased flow, with compensatory overdevelopment on the right side, most likely the result of increased flow. LAL was shown to decrease myocyte proliferation rates in LV (Sedmera et al., 2002). DeAlmeida et al. also showed that partial clipping of the right atrial appendage increases blood flow to LV leading to an increase in chamber volume and myocardial mass based on myocyte proliferation in both LAL and normal embryos (deAlmeida et al., 2007). Similarly, addition of fibroblast growth factor-2 (FGF2), a known stimulant of embryonic myocyte division was shown to increase cellular proliferation and abate the diseased phenotype (deAlmedia and Sedmera, 2009).

Effects of hemodynamic alterations from CTB and LAL on the developing conduction system were investigated by Reckova et al. (2003). CTB resulted in premature emergence of the mature apex-to-base activation sequence, whereas LAL resulted in delayed transition to a mature activation sequence. Biomechanics play a critical role in induction and patterning of the cardiac conduction system. Sedmera et al. studied the effects of hemodynamic alterations on the expression of endothelin converting enzyme (ECE) protein, which is involved in the inductive recruitment of Purkinje fibers in embryonic chick conduction systems (Sedmera et al., 2008). Following LAL, ECE expression was down-regulated in LVs with decreased preload and up-regulated in RVs with increased preload. Hearts cultured without hemodynamic loading showed decreased activation of the conduction pathway (Sankova et al., 2010). This primitive phenotype was rescued following artificial loading of the ventricles via a droplet of silicone oil. Together these findings suggest that loading is a necessary component of conduction system formation and maturation.

## Heart valve and outflow tract development

### Heart valve morphogenesis

During cardiac looping, TBX3 (Moorman et al., 2004) and Notch1 (Luna-Zurita et al., 2010) act to localize myocardial-endocardial signals to the AV and OFT regions. In the first stage of this process, a subset of endocardial cells lining these two zones transform into a mesenchymal phenotype and invade the cardiac jelly, a process known as endocardial to mesenchymal transformation (EMT) (Runyan and Markwald, 1983; Eisenberg and Markwald, 1995; Camenisch et al., 2002; Tavares et al., 2006; Butcher and Markwald, 2007; Combs and Yutzey, 2009). The invasive, proliferating mesenchyme progressively remodels the hyaluronan matrix, replacing it with proteoglycans, matricellular proteins, and eventually structural proteins such as collagen I (Norris et al., 2004; Person et al., 2005). These amorphous, compliant, cellularized masses, now dubbed cushions, continue to grow and extend into the lumen space (Schroeder et al., 2003)

Two cushions (superior and inferior) form initially in the AV canal at HH16 (E9.5 in mouse), followed by the appearance of two mural/lateral cushions on the left and right side of the AV canal at HH19 (Snarr et al., 2008). The superior and inferior AV cushions fuse together by HH26 (E12 in mouse) forming a septation of the AV canal that joins with the ventricular septum and the protruding atrial cap. The lateral portions of this fused mass undergo continual remodeling to form valves, as do the left and right mural cushions. During the remodeling of primitive AV valves into fibrous leaflets, the AV myocardium forms a fold at its junction with the ventricular myocardium creating a substrate on which the AV cushions can extend. The cushions extend along their substrate through the expansion of a proliferation zone in the subepithelial portion of the AV cushions (Sugi et al., 2003). Fenestrations develop as a result of the elongating cushions and the ventricular tissue underneath the cushion tissue delaminates, resulting in primitive leaflets that are continuous with developing papillary muscles and simultaneous expansion of the ventricular OFT (Wenink and Gittenberger-de Groot, 1986). The myocardial tissue of the AV valves disappears and they condense into fibrous leaflets (de Lange et al., 2004). Thin strands of elongated muscle remain tethered to the valve tissue with thickened trabecular aspects on the ventricular myocardial wall. These structures become the tendinous chords and papillary muscles of the mature valve (Icardo and Colvee, 1995). Epicardially derived fibroblasts have also been shown to contribute to the mural AV leaflets in developing murine heart**s** (Wessels et al., 2012).

The OFT differs in that endocardial cells along the entire lumen undergo EMT. Paired bulges emanating in proximal (just outside the right ventricle) and distal zones (just after a “dogleg” bend in the OFT) become cushions around HH22/E10, while the rest of the cardiac jelly regresses. The proximal/conal cushions are alternatively referred to as the septal/sinistroventral and parietal/dorsodextral ridges (Ya et al., 1998; Qayyum et al., 2001). A third distal cushion ridge subsequently forms (HH25/E11). These growing cushions fuse at the midline, forming two tortuous lumens. Between HH26/E11.5 and HH30/E13, the distal dorsal cushion of the OFT aligns with the proximal left cushion along the inner heart curvature, continuous with the superior cushion of the AV canal. Simultaneously, a wishbone shaped ridge of mesenchyme invades the OFT in a spiraling pattern, separating it into left and right portions and dividing the outflow cushions into two groups of three. While the fused proximal cushions myocardialize and form the muscular infundibulum, which separates the right and left ventricular outlets, the distal cushions become the rudiments of the pulmonary and aortic outlet valves (Qayyum et al., 2001). For the remodeling of primitive outflow valves into fibrous leaflets, unlike the AV valves, which formed through delamination from the muscular walls, valves of the OFT form through a process of excavation or hollowing of the cushion's aortic side. Cushion excavation begins at HH29/ED13 with a small depression in the arterial face of the cusps. The endothelium lining the aortic surface of the valves becomes thickened with rounded cells that flake and undergo apoptosis, while the ventricular epithelium remains flat and elongated (Garcia-Martinez et al., 1991). The deepening furrow condenses the fibrous matrix around it, creating thin cusps of tissue that are attached in an arc pattern called the commissures (Butcher and Markwald, 2007).

### Heart valve hemodynamics

**Figure 4:**
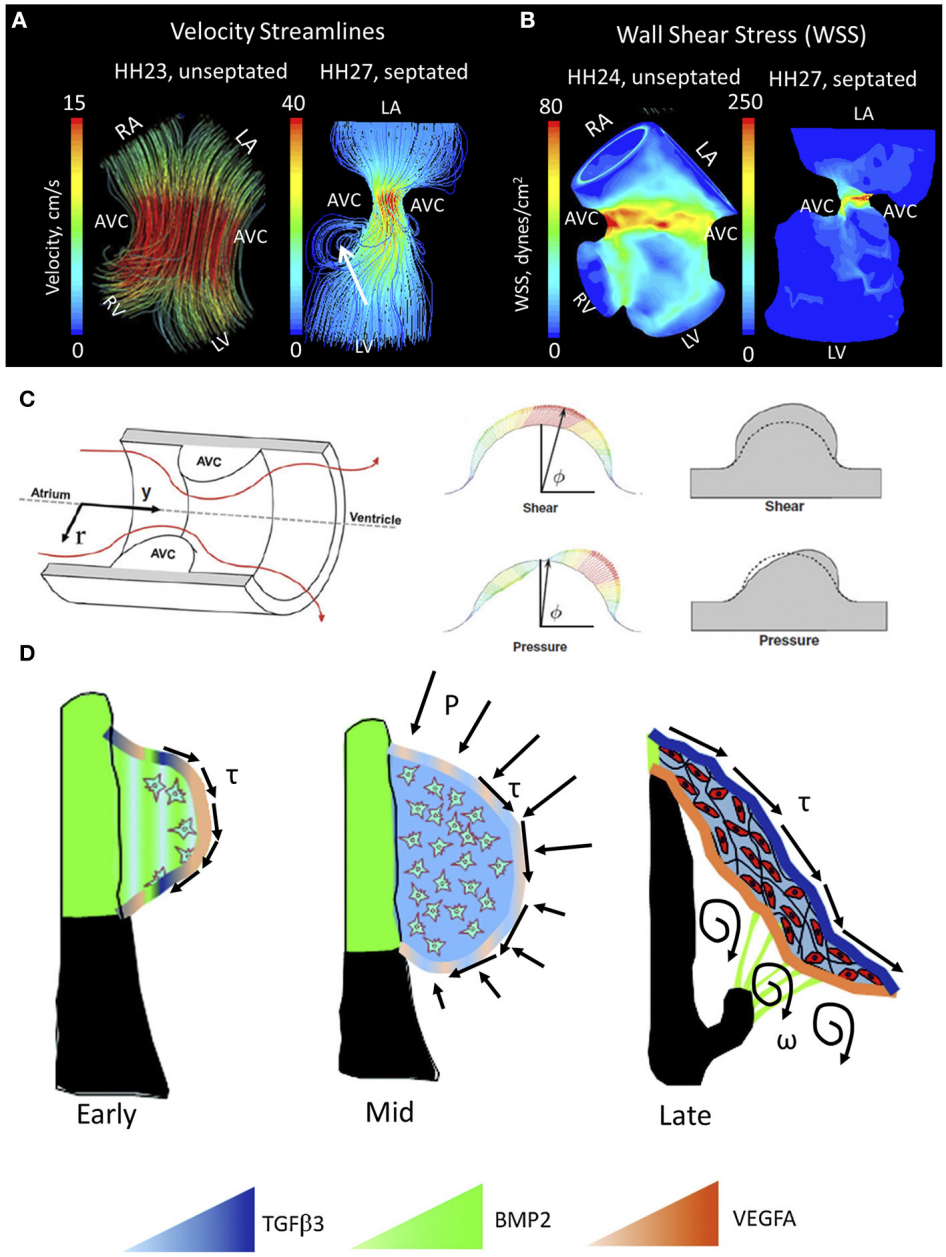
**Mechanical forces driving AV valve morphogenesis. (A)** Velocity streamlines in AV canal for HH23 (unseptated) and for HH27 (septated) chicken embryos, generated with computational fluid dynamics. In HH23, flow profile is laminar parallel streamlines, whereas in HH27 it is jet flow with circulating eddies beneath AV cushions (shown with arrrow). LA, left atria; RA, right atria; LV, left ventricle; RV, right ventricle; AVC, atrioventricular cushion **(B)** WSS levels for HH23 and HH27 embryos. High stress levels are localized to the mid cushion region. Adapted form Yalcin et al. (2011). **(C)** Hemodynamic-driven AV cushion growth and remodeling computational model. Based on this model, the pressure distribution on the AV cushion cause leaflet-like elongation in the direction of flow, whereas shear tractions regulated the remodeling of tissue near the cushion surface suggesting WSS is important in mechanotransduction. Adapted from Buskohl et al. (2012b). **(D)** Proposed AV valve morphogenesis model. WSS on cushion surface regulate gene expression for valve development. Pressure on the cushion surface cause leaflet-like elongation and circulatory flows beneath the cushion helps detachment of the leaflet from the myocardial wall. Growth factor expression levels were indicated via color gradation as shown with colored triangles. Adapted from Chiu et al. (2010). τ represents shear stress, P represents pressure, and ω represents circulations.

Blood flow guides cardiac morphogenesis, sculpting tissue by promoting growth in response to increased demands. Less than 48 h after incubation, the presence of two blood streams is apparent in the embryonic chicken heart. A spiraling complex is created, as the force of the larger stream pulls the smaller stream around it, changing the mechanical environment of the developing heart (Jaffee, 1965). The rapid growth of the endothelial tube inside the early heart is a result of an increase in blood pressure (Chang, 1932). In this way blood flow, shear stress and stretching forces are thought to influence the duration of vessel growth and their morphological characteristics (Taber and Eggers, 1996). During this time of heart formation, shear stress is greatest in the inner curvature and sites of lumen constrictions, corresponding to the AV canal and OFT where the ECs form and develop into functioning valves (Groenendijk et al., 2004). The velocity profile of flow through the primitive valves begins with a Poiseuille parabola (HH17), before resembling plug flow; cushions arise shortly after peak inflow velocity and extend perpendicularly to the direction of flow (Figures 4A,B, left) (Yalcin et al., 2011; Bharadwaj et al., 2012). As the heart continues to grow, the energy extended in pulsatile flow increases from one-third to two-thirds of total energy between HH18 and 29 (Clark et al., 1986). A jet profile characterizes the AV region of HH27 chick embryos (Figure 4A, right). Figure 4A, right, highlights circulating eddies, which form beneath the cushion surface. WSS levels increase from 80 dynes/cm^2^ at HH23 to 250 dynes/cm^2^ at HH27 (Figure 4B). As the embryo's pressure, cardiac output and WSS increase, the cushions elongate to form thin fibrous leaflets with increased ECM proteins and greater mechanical stiffness (Butcher et al., 2007; Biechler et al., 2010; Buskohl et al., 2012a). Strain energy density increases linearly with AV valve leaflet length (Buskohl et al., 2012a).

While the importance of biomechanics in the formation of the valve leaflets has been acknowledged for some time, only recently have individual roles for pressure and WSS been proposed. Buskohl et al. created a computational model in which a finite element model was coupled to a fluid dynamics model, highlighting interaction between the two (Figure 4C) (Buskohl et al., 2012b). Buskhol's iterative computational approach involved determining pressure and velocity profiles, transferring flow-induced pressure and shear tractions on the AV cushion surface, and updating the fluid simulations after inelastic deformation had taken place. Fluid shear tractions were found not to significantly alter cushion volume, but rather functioned as the driver for cushion surface remodeling. While pressure was found to be negligible in terms of tissue deformation, shear traction forces from the top-center cushion region greatly contributed to tissue elongation. Pressure was responsible for tissue resorption on the inflow side of AV cushions and expansion on the outflow side (Buskohl et al., 2012b). Regulation of the cushion surface, particularly the leading edge of valve leaflets, may be heavily influenced by mechanotransduction.

Altered hemodynamic flow patterns during critical periods of development have been shown to lead to a variety of cardiac abnormalities, many of which influence valve formation (Hogers et al., 1997; Sedmera et al., 1999; Hove et al., 2003). Maintenance of circulatory energy efficiency and pressure are critical for development (Lucitti et al., 2005). Sedmera et al. found that alterations in ventricular load influenced AV valve morphogenesis as well as trabeculation patterns (Sedmera et al., 1999). In embryos with increased ventricular afterload (CTB), right AV valve morphology no longer took the form of a muscular flap but rather resembled a bicuspid structure. Previous work by our group showed that photoablation of the superior AV cushions of HH24 chick embryos immediately altered AV canal hemodynamics (i.e., increased regurgitation), resulting in stunted ventricular and valvular growth in 48 h (Yalcin et al., 2010). Shear stress and shear stress-induced or repressed gene expression are important factors in remodeling of the cardiac cushions (Hove et al., 2003; Groenendijk et al., 2005; Vermot et al., 2009). Hove et al. attributed abnormal third chamber development in mechanically perturbed zebrafish embryos to a reduction in shear stress on ECs (Hove et al., 2003). Bartman et al. argue observed phenotypes were not the result of a decrease in shear alone. Their 2004 study found that 58% of zebrafish embryos treated with a myofibril inhibitor (2,3-butanedione monoxime) were still able to form an endocardial ring, affirming that blood flow is not required for the initial steps of cushion formation (Bartman et al., 2004).

### Hemodynamics and gene expression for valve development

While hemodynamics undoubtedly plays a large role in valvulogenesis, the role of hemodynamic signaling remains a point of contention. Though changes in shear stress have been found to presage the development of cardiac malformations (Hogers et al., 1997, 1999; Hove et al., 2003; Yashiro et al., 2007), other studies claim myocardial function surpasses shear stress as a major epigenetic factor (Bartman et al., 2004). Endocardial cells lining the luminal cushion surface may promote valvular morphogenesis by coupling mechanical stimuli and molecular signaling pathways (Butcher et al., 2007).

A number of studies have demonstrated the shear sensitivity of valvular morphogens *in vivo*. Endothelin-1 (ET-1) and endothelial nitric oxide synthase (NOS-3) are shear stress responsive genes linked to cardiovascular development by knockout mice that display a wide range of cardiovascular defects (Yanagisawa et al., 1998). Critical periods of cardiovascular remodeling (HH20–HH30) were marked by ET-1 and KLF2/NOS-3 restriction to narrow sites (Groenendijk et al., 2004). ET-1 was downregulated by shear stress, while KLF2 and NOS-3 were upregulated by shear. Observed patterns changed with vitelline vein obstruction and cardiovascular malformations ensued (Groenendijk et al., 2005). In addition to the aforementioned genes, the expression patterns of potent valvular morphogens, such as transforming growth factor β (TGFβ), bone morphogenetic protein (BMP), and vascular endothelial growth factor (VEGF) are spatially and temporally restricted in a manner that suggests hemodynamic regulation (Figure 4D) (Butcher and Nerem, 2007; Chiu et al., 2010). TGFβ appears in the endocardium of the valve forming regions around HH20, when flow transitions from its laminar poiseuille flow to more plug-like flow and rapidly increases in velocity (Yalcin et al., 2011). *In vitro* analyses suggest that TGFβ3 induces cell migration, invasion, and matrix condensation; BMP2 induces invasion; VEGFA inhibits invasion but increases migration (Tan et al., 2011). TGFβ3 was found to induce myofibroblastic differentiation in a dose-dependent manner, whereas VEGFA and BMP2 did not, suggesting that hemodynamic forces work through these transcription factors in the remodeling of the AV valves. Using an *in vitro* system, Tan et al. were able to show the flow-regulated development of the fibrous AV valves is dependent on rhoA expression (Tan et al., 2013). This body of work is depicted in Figure 4D.

### The role of hemodynamics in formation of in the great vessels

The great vessels are derived from the pharyngeal arch arteries. A total of six arch pairs sequentially emerge, regress, or remodel throughout OFT development. While arch arteries I and II ultimately regress, the third arch pair forms the mature brachiocephalic arteries. The mature aortic arch is derived from the right fourth arch in chicken and the left fourth arch in mammalian embryos (Wang et al., 2009). The sixth arch pair forms pulmonary artery and ductus arteriosus. The formation of the truncus arteriosus separates the pulmonary artery and right ventricular OFT from the aorta and left ventricular OFT posteriorly (Qayyum et al., 2001; Hu et al., 2009).

Hemodynamic forces initiate extensive remodeling of the symmetric aortic arch system in a highly asymmetric fashion (Hu et al., 2009). Yashiro et al. investigated the mechanisms behind regression of the right VI aortic arch and persistence of left VI arch in mice (Yashiro et al., 2007). Using a variety of experimental and genetic mutant models, they found that, the genetic programme, including the expression of Pitx2, induces a dynamic morphological change in the OFT, which in turn generates a differential distribution of blood flow. Mice lacking the asymmetric enhancer PITX2 failed to adopt the spiral structure of the OFT and in these embryos OFT remained linear. Changes in OFT geometry led to differential flow distribution, successive signaling abnormalities and further changes in geometry. In normal embryos, increased blood flow in the left VI arch sufficiently induces PDGFR and VEGFR2 signaling and subsequently maintains arterial structure. Decreased flow in the right VI arch artery leads to vessel regression. Wang et al. discovered that increases in WSS corresponded with increases in arch artery diameters in day 3 (HH18) and day 4 (HH24) chick embryos (Wang et al., 2009). CFD analysis revealed that HH21 embryos had elevated, rather than intermediate WSS compared to both HH18 and HH24 embryos (Kowalski et al., 2013) suggesting periods of vascular remodeling may be preceded by acute increases in WSS. Hu et al. showed that altered hemodynamic flow in the aortic arch arteries due to LAL was associated with a range of cardiac defects (Hu et al., 2009). Overall, these studies suggest hemodynamic forces play a significant role in asymmetric remodeling of aortic arch network. Characterization of aberrant hemodynamic flow may be a resource in understanding OFT and arch network abnormalities, which account for 50% of infants with a CHD (Roger et al., 2011).

## Hemodynamics and congenital heart defects

The embryonic heart adapts ventricular geometry and function to optimize mechanical efficiency (Lin and Taber, 1995). Distinctions between gene and hemodynamic-related abnormalities are not well defined. Though chromosomes linked to specific defects have been identified, they rarely provide a full picture, with only 10–15 percent of ventricular outflow malformations loosely associated to a chromosomal abnormality (McBride et al., 2009). While genetic mutations are associated with CHDs, genes cannot be manipulated to produce CHDs in the same way as flow can be perturbed to produce disease phenotypes. The use of computational modeling in the study of abnormal development has led to a more thorough analysis of disease etiologies and their underlying mechanisms. In this section, the pathology of two major CHDs associated with abnormal hemodynamic pattering and valve formation are explored: bicuspid aortic valve (BAV) and hypoplastic left heart syndrome (HLHS).

*BAV* is the most common congenital anomaly of the heart, marked by two aortic valve leaflets rather than three. This two cusped configuration constrains the patient, as the free edges of the bicuspid valve are more straight than round and offer limited mobility. The leaflets are usually of unequal size with a raphe, or seam-like union, apparent in the larger leaflet (Yener et al., 2002). BAV is frequently associated with aortic valve stenosis, regurgitation and endocarditis, though these symptoms develop well after valve formation. Excessive length of one or both cusps results in abnormal contact which in turn leads to fibrous thickening that will later become diffuse and calcified. Stenosis usually develops in bicuspid valves containing no redundant cusp tissue, while valve incompetence is associated with redundancy and endocarditis. The large calcific deposits associated with BAV are unusual before the age of 30 and very prevalent thereafter (Roberts, 1970). In a 2003 study of 44 bicuspid aortic valves, BAV patients without significant stenosis or regurgitation were found to have a larger aortic annulus, aortic sinus and proximal ascending aorta when compared to normal tricuspid valves. The peak aortic velocity (Nkomo et al., 2003) and peak systolic wall velocity in the anterolateral region of the ascending aorta (Bauer et al., 2006) were also found to be higher in BAV patients than controls. This flow has been classified as helical, or flow composed of a forward component along the long axis of the aorta and a rotational component, moving circumferentially along that same axis (Bissell et al., 2013; Lorenz et al., 2014). A significant increase in absolute peak helicity is present during systole of BAV patients, with a substantially greater distribution of mean helicity in the aorta (Lorenz et al., 2014).

Recent advances in cardiac imaging, have allowed scientists to map these regions and collect 3D spatial visualizations of flow patterns over time (Bissell et al., 2013; Lorenz et al., 2014). In this way, temporal evolution of complex flow patterns over time can be studied and linked to aortic function. Bissell et al. found that patients with BAV presented with predominantly abnormal right-handed helical flow in the ascending aorta, larger ascending aortas, higher helical flow, elevated systolic angle and elevated systolic WSS. In their study of 69 BAV patients, left-handed helical flow, normal flow, and complex flow occurred in 4, 11, and 13%, respectively, whereas right-handed helical flow occurred in 72% of patients. Although flow patterns differed, distensibility, aortic strain, and pulse wave velocity of the aorta were similar across all groups. Flow abnormalities initiate aortopathy as a means of maintaining optimal WSS values (Bissell et al., 2013). Through the use of fluid structure interaction models, (Chandra et al., 2012) degree of leaflet calcification is linked to orifice area, oscillatory shear index and temporal shear magnitude. While the regular tricuspid and non-coronary BAV leaflets shared similar shear stress characteristics, the base of fused BAV leaflet fibrosa differed greatly. The temporal shear index of fused leaflets was heavily modulated by degree of calcification, with 6- to 16-fold increases seen over BAVs ranging from normal to severely calcified. Results support a mechano-sensored model of calcified aortic valve disease in the BAV patients (Chandra et al., 2012).

*HLHS* is characterized by acute underdevelopment of the left ventricle. No strong genetic correlation exists. In a study of 83 HLHS patients, nine had underlying chromosomal abnormalities, four had single gene defects, 10 had one or more extracardiac anomaly and two were patients of insulin-dependent mothers (Natowicz et al., 1988). Disease formation is thought to result from diminished flow to the left ventricle and aortic OFT. Retrograde aortic flow may play a role in impaired development of the aortic root and ascending aorta (Simpson and Sharland, 1997). Cardiac defects associated with HLHS include mitral valve hypoplasia or mitral stenosis coincident with left heart obstruction, hypoplastic left ventricle, aortic atresia, hypoplastic aorta, and coarctation of the aorta. Out of 96 HLHS patients, 12.5 percent exhibited dysplastic aortic valvular stenosis, 37.5 percent were found to have malfunctioning aortic and mitral valves, 50 percent presented with abnormal AV valves (Ilbawi et al., 2007). To date, the LAL remains the only experimental model to fully recapulate these disease phenotypes (Sedmera et al., 1999, 2002; Tobita et al., 2002). Changes in WSS as shown by Kowalski et al. may be responsible for underdevelopment of left heart structures (Kowalski et al., 2013). Variations in heart rate and AV inflow velocity were acute to non-existent in these models (Tobita et al., 2002). Mechanical manipulation of ventricular filling dates back to Harh et al. (1973). Harh et al. inserted a nylon device into the left AV canal, thereby reducing ejection volume from the ventricle to ascending aorta (Harh et al., 1973). Failure of the cushions to differentiate into fibrous leaflets led to hypoplasia. Narrowing resulted in a reversed atrial shunt. Though Harh et al. observed disease phenotypes, they were unable to fully characterize mechanical changes. Combining experimental results and computational models presents a promising way of understand the mechanisms through which changes may arise.

## Conclusions and future directions

Mechanical forces are essential drivers of cardiac morphogenesis, transforming the linear heart tube into a multichambered unidirectional machine capable of adapting to environmental demands. CHDs arise when the heart is prevented from following the normal pathways of development; understanding the intricate mechanisms involved in heart development is necessary for the advancement of clinical solutions. The majority of CHDs result from improper positioning of the cardiac OFT, impaired remodeling of the cushions into valve leaflets, or abnormal remodeling of the arches into great vessels. Quantitative imaging modalities provide a gateway into elucidating CHD formation. Integrating these imaging modalities with CFD and growth modeling will greatly strengthen our inference of causative mechanical forces of CHDs, such as BAV and HLHS. Targeted surgical techniques, including laser ablation, can help scientists distinguish between the effects of flow and tissue deformation alone. *In vitro* 3D culture and bioreactor studies work to further our understanding of how specific mechanical forces influence multi-scale biological responses. As our insight into CHD etiology improves, so will our ability to effectively and targetedly restore proper remodeling of cardiac tissues.

### Conflict of interest statement

The authors declare that the research was conducted in the absence of any commercial or financial relationships that could be construed as a potential conflict of interest.
